# Association Between COVID-19 Infection and Cardiac Biomarkers in Hospitalized Patients at a Tertiary Care Center

**DOI:** 10.7759/cureus.41527

**Published:** 2023-07-07

**Authors:** Mahmoud W Bader, Abdulqader M Alaa Adeen, Omar E Hetta, Alwaleed K Aloufi, Muhannad H Fallata, Abdulaziz A Alsiraihi, Mohamed E Ahmed, Abdulhalim J Kinsara

**Affiliations:** 1 College of Medicine, King Saud Bin Abdulaziz University for Health Sciences, Jeddah, SAU; 2 College of Sciences & Health Professions, King Saud Bin Abdulaziz University for Health Sciences, Jeddah, SAU; 3 Cardiology, Ministry of National Guard - Health Affairs, Jeddah, SAU; 4 King Abdullah International Medical Research Center, Ministry of National Guard - Health Affairs, Jeddah, SAU

**Keywords:** risk factors, acute cardiac injury, beta natriuretic peptides (bnp), cardiac troponin, covid-19

## Abstract

Background

The effects of coronavirus disease 2019 (COVID-19) on the cardiovascular system are well established. However, knowledge gaps in the clinical implications of cardiac involvement in COVID-19 patients are yet to be addressed. This study aimed to investigate acute cardiac injury (ACI) risk factors and outcomes associated with COVID-19 infection with cardiac involvement.

Methodology

In this retrospective study, we included hospitalized patients between March 2020 and May 2022 with confirmed COVID-19 infection and evidence of cardiac involvement.

Results

In total, 501 patients were included, of whom 396 (79%) had evidence of ACI. The median troponin level was 25.8 (interquartile range (IQR) = 10.8-71). Patients with evidence of ACI were significantly more likely to have diabetes mellitus (75% vs. 60%), cardiovascular disease (48% vs. 37%), chronic lung disease (22.2% vs. 12.4%), and chronic kidney disease (32.3% vs. 16.2%). Additionally, patients with ACI were significantly more likely to have cardiomegaly (60.6% vs. 44.8%) and bilateral lobe infiltrates (77.8% vs. 60%) on X-ray. Patients with ACI were significantly more likely to suffer from complications such as cardiogenic shock (5.3% vs. 0%), pneumonia (80.1% vs. 65.7%), sepsis (24.2% vs. 9.5%), and acute respiratory distress syndrome (33.1% vs. 8.6%). Patients with ACI were also significantly more likely to be admitted to the intensive care unit (ICU) (57% vs. 26.7%) and significantly more likely to die (38.1% vs. 11.4%). The results of the multivariate regression analysis indicated that mortality was significantly higher in patients with elevated troponin levels (adjusted odds ratio = 4.73; 95% confidence interval = 2.49-8.98).

Conclusions

In COVID-19-infected patients, old age, diabetes mellitus, cardiovascular disease, chronic lung disease, and chronic kidney disease were associated with an increased risk of ACI. The presence of ACI in the context of COVID-19 infection was noted to increase the risk for severe complications, such as cardiogenic shock, ICU admission, sepsis, and death.

## Introduction

Coronavirus disease 2019 (COVID-19) is caused by the novel severe acute respiratory syndrome coronavirus 2 (SARS-Cov-2) which mainly affects the respiratory system. However, cardiac involvement in COVID-19 is a significant cause of morbidity and mortality. The mechanism of cardiac injury in COVID-19 is thought to be caused by the binding of SARS-CoV-2 to angiotensin-converting enzyme 2 (ACE-2) receptors on the cardiac myocytes. Another proposed mechanism is a cytokine storm caused by systemic inflammation and enhanced by elevated angiotensin 2 levels [1-3]. Various cardiac complications have been reported, such as myocarditis, cardiac injury, cardiac infarction, heart failure, arrhythmias, and thromboembolic events. These complications have been associated with increased morbidity and mortality [4,5]. COVID-19 can cause changes in both cardiac biomarkers and imaging. Elevated cardiac biomarkers, such as cardiac troponin and b-type natriuretic peptide (BNP), have been reported in hospitalized COVID-19 patients [6]. Additionally, previous studies have demonstrated that specific X-ray findings, such as cardiomegaly and air space infiltrates, are associated with an increased risk of acute cardiac injury (ACI) [7]. Several studies have also reported a correlation between right ventricle dilation and disease severity [8-10].

There is a lack of detailed analysis of the relationship between COVID-19 infection and cardiac manifestations in the literature. This study aimed to analyze the association between elevated cardiac biomarkers and the severity of COVID-19 infection.

An earlier version of this article was posted on the Research Square preprint server on May 15, 2023.

## Materials and methods

This retrospective cohort study was performed at a tertiary care center in Saudi Arabia. The study included hospitalized COVID-19 patients with signs of cardiac involvement from March 2020 to May 2022. Cardiac involvement was defined as either abnormality in high-sensitivity cardiac troponin I (hs-cTnI), BNP, electrocardiogram (ECG), or echocardiogram. The indications for admission were severe hypoxia, defined as SpO_2_ below 90%, or the presence of comorbidities and clinical instability.

The BestCare 2.0 system, an electronic health record system, was used to collect the data retrospectively. Before conducting the study, the Institutional Review Board (IRB) approval was obtained from King Abdullah International Medical Research Center (approval number: IRB/1180/22).

COVID-19 was diagnosed with a positive real-time reverse transcription polymerase chain reaction (RT-PCR) or positive serology for anti-SARS-COV-2 specific IgM or IgG antibodies. Patients were categorized into two groups, according to the hs-cTnI levels. Patients with a hs-cTnI level above the 99th percentile were designated as the ACI group, and those with a hs-cTnI below the 99th percentile were designated as the non-ACI group. An abnormal BNP was defined as a level of more than 100 pg/mL. The parameters used in this study were patient demographics, comorbidities, risk factors, laboratory findings, imaging findings, ECG findings, intensive care unit (ICU) admission, management, and complications.

The SPSS software version 28 (IBM Corp., Armonk, NY, USA) was used to analyze the data. The descriptive statistics are presented as categorical and numerical variables. The categorical data are presented as frequency, proportions, and bar charts, and the numerical data as frequency tables and histograms. Mean and standard deviation was used for normally distributed data, while median and interquartile range (IQR) were used for skewed data. Inferential statistics are presented as categorical and numerical variables. The chi-square or Fisher’s exact test was used to compare the categorical data. For normally distributed numerical data, Student’s t-test was used. A median test (Mann-Whitney) was used for skewed numerical data distribution. Risk factors were analyzed using regression analysis. Statistical significance was set at p-value <0.05. A multivariate regression analysis model was created to investigate the risk factors associated with ACI adjusting for age, gender, diabetes, and chronic kidney disease. Additionally, a second model was created to analyze the risk factors associated with in-hospital mortality adjusted for age and gender.

## Results

A total of 2,421 patients were hospitalized due to confirmed COVID-19 infection, of whom 501 (20.7%) had cardiac involvement. Of the 501 patients, 396 (79%) had evidence of ACI. Just more than half (n = 272, 54.3%) were male. The mean age was 67.5 (SD = 15.6) years. The median total duration of hospital admission was 13 days (IQR = 7-26 days), and the median duration of ICU admission was one day (IQR = 0-13).

There was no significant difference between the prevalence of ACI between males and females. The mean age of the group with ACI was significantly higher compared to the group without ACI (mean = 68.7 vs. 63.1; p = 0.003). The ACI group had a significantly longer total length of hospital stay (median = 15 vs. 9; p < 0.001) and length of ICU stay (median = 4 vs. 0; p < 0.001) (Table 1).

**Table 1:** Patient demographics. ACI = acute cardiac injury; ICU = intensive care unit; SD = standard deviation; IQR = interquartile range

	Total (n = 501)	Non-ACI (n = 105)	ACI (n = 396)	P-value
Male gender, n (%)	272 (54.3)	63 (60)	209 (52.8)	0.185
Age, mean years (SD)	67.5 (15.6)	63.1 (19.2)	68.7 (14.3)	0.003
Total duration of hospital admission, median days (IQR)	13 (7–26)	9 (5.5–17.5)	15 (8–27)	<0.001
Duration of ICU admission, median days (IQR)	1 (0–13)	0 (0–1)	4 (0–15)	<0.001

Overall, risk factors such as diabetes mellitus (75.3% vs. 60%; p = 0.003), known cardiovascular disease (48.2% vs. 37.1%; p = 0.042), chronic kidney disease (32.3% vs. 16.2%; p < 0.001), and chronic lung disease (22.2% vs. 12.4%; p = 0.02) were significantly more prevalent in patients with ACI. No significant difference between the two groups was noted regarding smoking, hypertension, cerebrovascular disease, heart failure, malignancy, vaccination status, body mass index, HbA1c, or dyslipidemia (Table 2).

**Table 2:** Risk factors Variables are represented as numbers and frequencies unless otherwise stated. ACI = acute cardiac injury; BMI = body mass index; SD = standard deviation; IQR = interquartile range

	Total (n = 501)	Non-ACI (n = 105)	ACI (n = 396)	P-value
BMI, mean (SD)	29.9 (7.60)	30 (7)	29.9 (7.8)	0.579
Diabetes mellitus	361 (72.1)	63 (60)	298 (75.3)	0.003
HbA1c, median (IQR)	7.2 (6.2–9.1)	6.8 (5.9–8.8)	7.3 (6.2–9.1)	0.212
Hypertension	364 (72.7)	72 (68.6)	292 (73.7)	0.296
Cardiovascular disease	230 (45.9)	39 (37.1)	191 (48.2)	0.042
Cerebrovascular disease	99 (19.8)	28 (26.7)	71 (17.9)	0.052
Heart failure	119 (23.8)	21 (20)	98 (24.8)	0.303
Dyslipidemia	125 (25.0)	28 (26.7)	97 (24.5)	0.649
Smoking	44 (8.8)	14 (13.3)	30 (7.6)	0.077
Malignancy	77 (15.4)	19 (18.1)	58 (14.7)	0.392
Chronic kidney disease	145 (29.0)	17 (16.2)	128 (32.3)	<0.001
Chronic lung disease	101 (20.2)	13 (12.4)	88 (22.2)	0.02
Not vaccinated	468 (93.4)	100 (95.2)	368 (92.9)	0.581
One dose	17 (3.4)	2 (1.9)	15 (3.8)	
Two doses	16 (3.2)	3 (2.9)	13 (3.3)	

The group without ACI presented more frequently with vomiting than patients who suffered ACI (21.9% vs. 13.6%; p = 0.044). Other symptoms, including fever, cough, dyspnea, diarrhea, or muscle aches, did not have an association with ACI.

Patients with ACI had a higher incidence of bilateral lobe infiltrates on chest X-ray (77.8% vs. 60%; p < 0.001) and cardiomegaly (60.6% vs. 44.8%; p = 0.004). No difference was noted in the occurrence of pulmonary edema or pleural effusion. The major findings on the echocardiogram were left ventricular dysfunction, mitral valve regurgitation, right ventricular dilation, and right ventricular dysfunction; none of these findings showed significant differences between patients with ACI and patients without ACI. The two major findings on CT of the chest were ground-glass opacity and consolidation. ECG findings included bundle branch block, ST elevation, QTc prolongation, atrial fibrillation, and T-wave inversion. These findings were more common in patients with ACI, although the difference was not statistically significant (Table 3).

**Table 3:** Clinical presentation and imaging. All variables are represented as numbers and frequencies. ACI = acute cardiac injury; ECG = electrocardiograph; QTc = corrected QT interval; CT = computed tomography

	Total (n = 501)	Non-ACI (n = 105)	ACI (n = 396)	P-value
Symptoms				
Fever	289 (57.7)	64 (61)	225 (56.8)	0.445
Cough	293 (58.5)	58 (55.2)	235 (59.3)	0.449
Dyspnea	312 (62.3)	66 (62.9)	246 (62.1)	0.89
Diarrhea	70 (14.0)	13 (12.4)	57 (14.4)	0.592
Vomiting	77 (15.4)	23 (21.9)	54 (13.6)	0.044
Muscle ache	64 (12.8)	18 (17.1)	46 (11.6)	0.144
ECG findings				
Bundle branch block	29 (5.8)	5 (4.8)	24 (6.1)	0.605
ST-elevation	17 (6.4)	1 (2)	16 (7.9)	0.098
QTc prolongation	38 (14.9)	9 (18)	29 (14.2)	0.501
Atrial fibrillation	63 (23.8)	8 (16)	55 (25.6)	0.138
T-wave inversion	34 (6.8)	7 (6.7)	27 (6.8)	0.956
X-ray findings				
Bilateral infiltrates	371 (74.1)	63 (60)	308 (77.8)	<0.001
Pleural effusion	227 (45.3)	43 (41)	184 (46.5)	0.312
Cardiomegaly	287 (57.3)	47 (44.8)	240 (60.6)	0.004
Pulmonary edema	89 (17.8)	16 (15.2)	73 (18.4)	0.44
CT findings				
Ground glass opacity	75 (15.0)	15 (14.3)	60 (15.2)	0.824
Consolidation	47 (9.4)	6 (5.7)	41 (10.4)	0.127
Echocardiogram findings				
Right ventricular dilation	14 (2.8)	3 (2.9)	11 (2.8)	0.965
Right ventricular dysfunction	37 (7.4)	1 (1)	15 (3.8)	0.096
Left ventricular dysfunction	16 (3.2)	8 (7.6)	29 (7.3)	0.918
Mitral valve regurgitation	31 (6.2)	5 (4.8)	26 (6.6)	0.483

Patients with ACI had higher respiratory rates and lower peripheral capillary oxygen saturation. Additionally, patients with ACI had a higher white blood cell (WBC) count, higher procalcitonin, abnormal liver function test, and more prolonged partial thrombin time (Table 4).

**Table 4:** Vitals and laboratory findings. All variables are represented as median and interquartile range. ACI = acute cardiac injury; SBP = systolic blood pressure; HR = heart rate; RR = respiratory rate; SpO_2_ = peripheral oxygen saturation; WBC = white blood cell; ESR = erythrocyte sedimentation rate; Hgb = hemoglobin; eGFR = estimated glomerular filtration rate; ALT = alanine aminotransferase; NT-proBNP = n-terminal pro type-b natriuretic peptide; Hs-cTnI = high-sensitivity cardiac troponin I; AST = aspartate aminotransferase; PTT = partial prothrombin time; LDH = lactate dehydrogenase

	Total (n = 501)	Non-ACI (n = 105)	ACI (n = 396)	P-value
HR (bpm)	90 (80–103)	88 (76.5–102)	91 (81.3–103)	0.113
SBP (mmHg)	128 (113–145.5)	125 (109–145)	130 (114.3–146)	0.481
RR (bpm)	23 (20–28)	22 (20–27)	23 (20–30)	0.012
SpO_2_ (%)	95 (91–98)	97 (93–99)	95 (90–98)	0.004
WBC (×10^9^/L)	6.6 (4.6–9.4)	5.7 (4.3–8.7)	6.7 (4.8–9.6)	0.023
lymphocyte count (×10^9^/L)	1.11 (0.76–1.64)	1.105 (0.8–1.6)	1.11 (0.7–1.6)	0.989
Hgb (g/dL)	12.1 (10.4–13.7)	12.1 (9.8–13.7)	12.1 (10.6–13.7)	0.441
platelet count (×10^9^/L)	216 (157–286)	229.5 (159.3–287.5)	212 (157–286)	0.256
Procalcitonin (μg/L)	0.18 (0.08–0.62)	0.1 (0.1–0.2)	0.22 (0.1–0.7)	<0.001
ESR (mm/hour)	79 (53–109)	65 (42–106.5)	80.5 (57–111)	0.313
eGFR (mL/minute/1.73m^2^)	51 (23.75, 86.25)	74 (48–95)	48 (23–85)	0.071
Albumin (g/L)	35 (33–39)	36 (33–39)	35 (32–39)	0.228
AST (IU/L)	34 (24–52)	27 (20–41)	36 (25–54)	<0.001
ALT (U/L)	23 (15–39)	21 (14–36)	24 (16–40)	0.102
LDH (U/L)	350 (264–488.75)	315 (222–403)	377 (274–513)	<0.001
Hs-cTnI (pg/mL)	25.8 (10.8–71)	8.2 (3.6–14.5)	35.7 (15.5–90.3)	<0.001
Highest recorded Hs-cTnI	97 (34.8–381.95)	9.8 (4.6–18.7)	140.8 (56.9–617.6)	<0.001
NT-proBNP (pg/mL)	131.5 (56–376.25)	132 (62–256)	128 (56–515)	0.365
Creatine kinase (IU/L)	97 (47–215)	76 (32–187)	101 (51–221)	0.043
D-dimer (mg/L)	1.18 (0.6–2.57)	0.97 (0.5–2.9)	1.2 (0.6–2.5)	0.158
PTT (seconds)	33 (29–37)	33 (28–36)	34 (29.8–38)	0.008

Patients with ACI were significantly more likely to require mechanical ventilation (55.6% vs. 21.9%; p < 0.001) and receive systemic steroids (75% vs. 64.8%; p = 0.004). Patients with evidence of ACI were more likely to suffer from complications such as pneumonia (80% vs. 65.7%; p = 0.003), acute respiratory distress syndrome (33.1% vs. 8.6%; p < 0.001), complete picture of sepsis (24.2% vs. 9.5%; p < 0.001), arrhythmias (42% vs. 30.5%; p = 0.22), and cardiogenic shock (5.3% vs. 0%; p = 0.001). In addition, patients with ACI were more likely to be admitted to the ICU, more likely to suffer from cardiac arrest, and more likely to die (57.1% vs. 26.7%, 38.9% vs. 13.3%, and 38.1% vs. 11.4%, respectively; p < 0.001) compared to patients with no evidence of ACI. The median total duration of hospital admission (15 days; IQR = 8-27 vs. 9 days; IQR = 5.5-17.5; p < 0.001) and median duration of ICU admission (4 days; IQR = 0-15 vs. 0 days IQR = 0-1; p < 0.001) were significantly longer among ACI patients (Table 5).

**Table 5:** Management and complications. All variables are represented as numbers and frequencies. ACI = acute cardiac injury; ARDS = acute respiratory distress syndrome; ADHF = acute decompensated heart failure; ICU = intensive care unit

	Total (n = 501)	Non-ACI (n = 105)	ACI (n = 396)	P-value
Mechanical ventilation	243 (48.5)	23 (21.9)	220 (55.6)	<0.001
Systemic steroids	365 (72.9)	68 (64.8)	297 (75)	0.04
Immunoglobulin	24 (4.8)	7 (6.7)	17 (4.3)	0.33
Acute kidney injury	153 (30.5)	0 (0)	21 (5.3)	<0.001
Cardiogenic shock	21 (4.2)	0 (0)	21 (5.3)	0.001
Myocarditis	8 (1.6)	0 (0)	8 (2)	0.214
Thromboembolism	52 (10.4)	9 (8.6)	43 (10.9)	0.486
Arrhythmias	201 (40.1)	32 (30.5)	169 (42.7)	0.022
Pneumonia	386 (77.0)	69 (65.7)	317 (80.1)	0.003
Sepsis	106 (21.2)	10 (9.5)	96 (24.2)	<0.001
ARDS	140 (28)	9 (8.6)	131 (33.1)	<0.001
ADHF	16 (3.2)	6 (6.1)	10 (2.6)	0.105
ICU admission	254 (50.7)	28 (26.7)	226 (57.1)	<0.001
Cardiac arrest	168 (33.5)	14 (13.3)	154 (38.9)	<0.001
Death	163 (32.5)	12 (11.4)	151 (38.1)	<0.001

The results of the multivariate analysis show that elevated aspartate aminotransferase (AST), creatine kinase, and D-dimer were associated with an increased risk of ACI (adjusted odds ratio (OR) = 3.14, 1.05, and 1.51, respectively). In addition, cardiomegaly on X-ray was significantly associated with ACI (adjusted OR = 1.73; 95% confidence interval (CI) = 1.11, 2.72) (Table 6).

**Table 6:** Univariate and multivariate regression analysis of the risk factors associated with cardiac injury ^1^Adjusted for age, gender, diabetes, and chronic kidney disease. OR = odds ratio; BNP = b-type natriuretic peptide; AST = aspartate aminotransferase

	Unadjusted OR (95% CI)	P-value	Adjusted OR^1^ (95% CI)	P-value
Elevated BNP	0.79 (0.51, 1.22)	0.291	0.66 (0.42, 1.04)	0.070
Elevated AST	2.46 (1.56, 3.87)	<0.001	3.14 (1.94, 5.10)	<0.001
Elevated creatine kinase	1.35 (0.80, 2.29)	0.265	1.05 (0.66, 1.67)	0.823
Elevated D-dimer	1.58 (1.02, 2.46)	0.042	1.51 (0.96, 2.38)	0.076
Cardiomegaly on X-ray	1.90 (1.23–2.94)	0.004	1.73 (1.11, 2.72)	0.016

The second multivariate analysis model results showed that elevated troponin, AST, D-dimer, and lactate dehydrogenase (LDH) were significantly associated with increased mortality risk (adjusted OR = 4.73, 2.72, 1.79, and 2.3, respectively) (Table 7).

**Table 7:** Univariate and multivariate regression analysis of the risk factors associated with in-hospital mortality. ^1^Adjusted for age and gender. OR = odds ratio; Hs-cTnI = high-sensitivity cardiac troponin I; BNP = brain natriuretic peptide; AST = aspartate aminotransferase; LDH = lactate dehydrogenase

	Unadjusted OR (95% CI)	P-value	Adjusted OR^1 ^(95% CI)	P-value
Elevated Hs-cTnI	4.77 (2.53, 9.00)	<0.001	4.73 (2.49, 8.98)	<0.001
Elevated BNP	0.83 (0.57, 1.21)	0.340	0.79 (0.54, 1.15)	0.220
Elevated AST	2.78 (1.88, 4.10)	<0.001	2.72 (1.83, 4.04)	<0.001
Elevated creatine kinase	1.38 (0.90, 2.11)	0.140	1.30 (0.84, 2.01)	0.245
Elevated D-dimer	1.79 (1.18, 2.72)	0.006	1.79 (1.17, 2.72)	0.007
Elevated LDH	2.34 (1.52, 3.61)	<0.001	2.30 (1.50, 3.60)	<0.001

**Figure 1:**
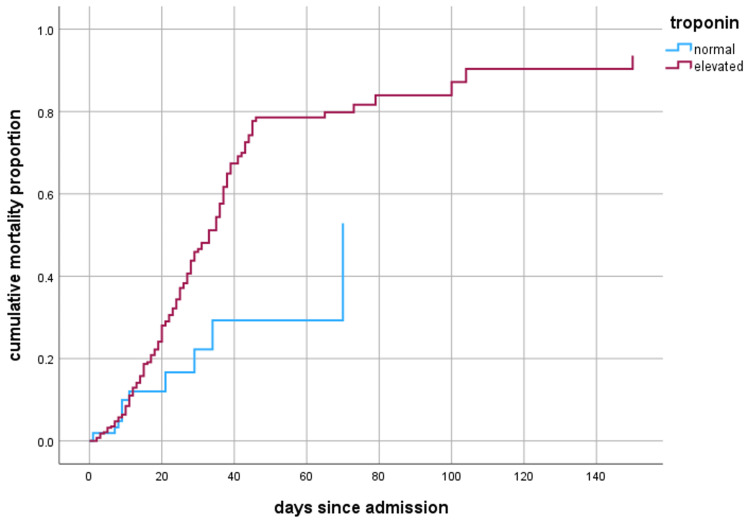
Cumulative in-hospital mortality proportions according to the value of cardiac troponin. Compared to patients with normal troponin levels throughout the admission, patients with elevated troponin had a higher mortality rate.

The Kaplan-Meier method was used to estimate the cumulative mortality proportions for troponin. Patients were divided into “normal” and “elevated” based on the levels of troponin. Patients with ACI had significantly shorter median survival duration than patients without ACI (70 vs. 33 days; p = 0.001). Additionally, the hazard ratio for troponin adjusted for age and gender was 2.11 (p = 0.013) (Figure 1).

## Discussion

The study found that 20.7% of the hospitalized COVID-19 patients had cardiac involvement, with 79% showing evidence of ACI. Our results suggest that certain risk factors are associated with an increased likelihood of developing cardiac injury in COVID-19 patients, including diabetes mellitus, a history of cardiovascular disease, chronic kidney disease, and chronic lung disease. Additionally, mortality was linked to an elevated troponin, AST, and D-dimer, as reported in previous studies. In line with our findings, multiple studies have reported an increased prevalence of cardiac injury in older patients with COVID-19 and patients with multiple risk factors [11-14]. The literature reported a significant increase in the prevalence of comorbidities in COVID-19 patients with cardiac injury, namely, hypertension, diabetes mellitus, chronic kidney disease, and chronic obstructive pulmonary disease [13,15,16]. In addition, a study showed that COVID-19 patients with more than two of the comorbidities are susceptible to developing an acute myocardial injury [17]. Although hs-cTn is an extremely sensitive biomarker for myocardial injury, it may identify troponin elevation above the reference threshold in non-cardiac conditions, such as renal dysfunction [18]. However, to ensure that the elevation of troponin is not originating from non-cardiac causes, this study followed The Fourth Universal Definition of Myocardial Infarction guideline that defined myocardial injury as an elevation of the troponin levels above the 99th percentile despite underlying causes [19].

The clinical manifestations of COVID-19 were similar to those reported in the literature, with the main symptoms including fever, dyspnea, and cough [20]. In line with our findings, multiple studies suggest that an elevated troponin correlates with an increased risk of morbidity and mortality [11-13,21,22]. Moreover, previous studies also highlighted the association between ACI and elevated LDH, WBC, and procalcitonin, which might correlate with the severity of systemic inflammation, cytokine storm, and tissue necrosis, leading to multiorgan dysfunction, including the heart [15,23,24]. Additionally, previous studies also reported a higher AST and longer prothrombin time in patients with ACI compared to patients with no evidence of ACI [13,23].

In line with the literature, bilateral infiltrates and cardiomegaly were associated with an increased risk of ACI [15,17,24,25]. However, the findings of other imaging modalities, such as echocardiography and CT, showed no association with ACI.

The use of mechanical ventilation and systemic steroid use was significantly more common in ACI patients, as reported [12,15,26]. In this study, severe complications such as acute kidney injury, cardiogenic shock, arrhythmias, ICU admission, and death were significantly higher in ACI patients, as reported in the literature [16,24,27].

This study identified elevated troponin, AST, and LDH as independent risk factors for mortality. Previous studies have also demonstrated the association between elevated troponin and a higher risk of acute kidney injury, acute respiratory distress syndrome, ICU admission, and a higher mortality rate. However, it is unclear whether the risk correlates with the troponin concentration. These studies also showed that increased levels of inflammatory markers are associated with a poor prognosis [16,23,28].

When interpreting the results of this study, the following limitations should be considered. This study was retrospective and conducted at a single center, which may limit the generalizability of our findings to other populations. In the early stages of the pandemic, all patients who tested positive for COVID-19 were hospitalized for isolation, which may have led to a lower prevalence of cardiac involvement in hospitalized patients. Despite these limitations, our study provides important insights into the risk factors and outcomes associated with cardiac involvement in COVID-19 patients. Future research is needed to identify effective strategies for managing cardiac involvement in COVID-19 patients.

## Conclusions

Infected COVID-19 patients who had ACI were older and had higher rates of diabetes mellitus, cardiovascular disease, chronic lung disease, and chronic kidney disease. On presentation, they had a higher rate of gastrointestinal symptoms, lung infiltrates, and cardiomegaly. Clinically, they were noted to be more hypoxic, with higher WBC, procalcitonin, and partial prothrombin time. They were more likely to develop arrhythmia, acute kidney injury, sepsis, cardiogenic shock, and cardiac arrest. Additionally, they were more likely to be admitted to the ICU, more likely to require mechanical ventilation, and had higher mortality rates. It is essential to carefully monitor COVID-19 patients for the risk factors that may increase the likelihood of cardiac involvement. Further research is needed to understand the mechanisms underlying cardiac involvement in COVID-19 patients and to identify effective treatment strategies.
